# Convergent and Known-Groups Validity and Sensitivity to Change of the Virtual Performance Measure in Patients With Hip and Knee Osteoarthritis: Longitudinal Study

**DOI:** 10.2196/69001

**Published:** 2025-03-28

**Authors:** Helen Razmjou, Suzanne Denis, Susan Robarts, Amy Wainwright, Patricia Dickson, John Murnaghan

**Affiliations:** 1 Sunnybrook Health Science Centre University of Toronto Toronto, ON Canada

**Keywords:** virtual, video-based outcome, longitudinal validity, sensitivity to change, osteoarthritis

## Abstract

**Background:**

Subsequent to the COVID-19 pandemic in 2020, a different approach to health care utilization was required to improve safety and efficiency. In the postpandemic era, virtual care and remote assessment of musculoskeletal conditions has become more common, and examining the accuracy of these remote encounters remains vital. In 2023, an innovative, video-based tool—the Virtual Performance Measure (VPM)—was introduced to assess the functional difficulties of patients with osteoarthritis of the knee joint. Further validation of this tool is warranted to expand its application longitudinally and in more diverse populations.

**Objective:**

This study examined the longitudinal validity of the VPM, a digitally based outcome tool, in patients with osteoarthritis of the hip and knee joints who had undergone arthroplasty.

**Methods:**

Patients completed a web-based survey after watching 40 videos that demonstrated 10 functional tasks with increasing difficulty, prior to and at approximately 3-5 months following surgery. The Lower Extremity Functional Scale (LEFS) was used as the reference measure. Longitudinal convergent and known-groups validity as well as sensitivity to change were assessed.

**Results:**

The data of 120 patients (n=80, 67% female; mean age 67, SD 9 years; n=58, 48% with hip osteoarthritis and n=62, 52% with knee osteoarthritis) were examined. There was a statistically significant improvement in both LEFS (*t*_119_=16.04, *P*<.001) and VPM total scores (*t*_119_=13.92, *P*<.001) over time. The correlation between the postoperative LEFS and VPM scores was higher (*r*=0.66; *P*<.001) than the correlation between the change scores of these measures (*r*=0.51; *P*<.001). The area under the curve value for the VPM’s ability to differentiate between urgent and nonurgent candidates for surgery was 0.71 (95% CI 0.57-0.84). Sensitivity to change as measured by the standardized response mean was 1.27 (95% CI 1.09-1.45), indicating good ability to detect change over time.

**Conclusions:**

The VPM demonstrated sufficient longitudinal convergent and known-groups validity as well as sensitivity to change in patients with hip and knee osteoarthritis following arthroplasty. This tool has a potential to improve the delivery of care by increasing access, reducing the frequency of in-person visits, and improving the overall efficiency of the health care system following a major surgery.

## Introduction

Osteoarthritis of the hip and knee joint is a disabling condition in older adults with significant physical, social, and economic burdens. Traditionally, two types of outcome measurement have been used in the assessment of patients with lower-extremity osteoarthritis. Patient-reported outcome measures document perceived physical difficulty within a specific timeframe [1]. Performance-based measures reflect the actual ability of the patients to execute a specific physical activity at the time of assessment [2]. The first video-based outcome measure that permitted a remote assessment of functional difficulties was introduced by a team of Dutch investigators in 2014 using videos with animated figures [3-5]. Subsequent to the COVID-19 pandemic in 2020, a different approach to health care utilization was required to improve safety and efficiency. In 2023, a next-generation, innovative, virtual assessment tool—the Virtual Performance Measure (VPM)—was introduced [6]. The VPM replaced animated figures with human models and combined features of self-report and performance-based tools, creating an outcome measure that could be used remotely. The original studies by the developers have shown cross-sectional validity of the VPM in patients with hip and knee joint osteoarthritis [6,7]. As clinicians continue to face challenges in care delivery and digital technology is being adopted more often by older persons in the postpandemic era, further validation of this tool is warranted to expand its application longitudinally and in more diverse populations.

The objective of this study was to examine the longitudinal convergent construct and known-groups validity as well as sensitivity to change of the VPM total score in patients with osteoarthritis of the hip and knee joints who had undergone joint replacement surgery. We explored the role of age, sex, and severity score on the postoperative VPM score after accounting for the preoperative VPM score. We hypothesized that (1) the postoperative and change scores of the VPM would correlate moderately with those of a traditional, validated, self-report functional tool; (2) the VPM would differentiate between patients who were considered urgent versus nonurgent for joint arthroplasty; and (3) the VPM score would show a large sensitivity to change following surgery.

## Methods

### Participants

This was a longitudinal study. All patients were recruited at a rapid-access clinic of a large arthroplasty center of an academic hospital in Canada. Inclusion criteria were failed nonsurgical management and candidacy for primary elective arthroplasty. Exclusion criteria included inability to read English, presence of cognitive conditions, lack of access to internet, or inability to use web-based surveys.

### Ethical Considerations

The study protocol was approved by the Human Ethics Research Board of the Sunnybrook Health Sciences Centre (Project# 3703). All patients provided informed consent for participation in the study. The web-based survey used a participant number, no personal identifiers were collected, and study data were anonymous. Patients were not compensated for their participation in the study.

### VPM Procedure

Patients who agreed to participate in the study received a link from a research assistant to a web-based survey. After entering their participant number, they completed the survey after watching 40 videos of 10 tasks on a smart phone, computer, or tablet twice—prior to surgery and 3-5 months following surgery. Activities varied from simple tasks such as sitting on a chair to physically demanding tasks such as navigating stairs, to provide a wide variety of activities and avoid ceiling and floor effects. The activities and their variations were selected based on input from physical therapists, occupational therapists, and patient representatives. The final set of the videos used an age-appropriate model.

The videos represented 10 commonly executed functional tasks with 4 levels of difficulty (normal to severe). Patients had an option of choosing “unable to perform” if the task was considered impossible to perform. The daily tasks included sitting and rising from a chair, putting on or taking off socks, getting into the shower, picking up an item from the floor, sitting down and getting up from the floor, walking on even ground, and ascending and descending stairs. The survey results were sent to the researchers via a secure platform. Further details on the development and cross-sectional validity of the tool have been provided elsewhere [6,7]. The videos are available to public (patients and researchers) [8].

### Preoperative Assessment

Initial assessment was conducted by advanced practice providers, who assigned a score to each candidate using the Severity Scoring System (SSS). The SSS is a validated scoring system and acts as a guide to interpreting findings across three main elements of the standardized assessment: (1) patient’s clinical presentation, (2) self-report pain and functional difficulties, and (3) severity of osteoarthritis based on imaging. The score helps improve access and outcomes in candidates for joint arthroplasty by determining an appropriate management plan, including assigning urgency and priority levels for surgery [6,7,9,10]. The SSS has been aligned with the Wait Time Information System priority levels and classifications in Canada [11]. The clinical component score of the SSS ranges from 0-3, the functional component score varies from 0-3, and the radiological examination score varies from 0-2. The total severity score ranges between 0-8, with higher numbers indicating a higher severity. Patients with a score of 7 or higher are considered urgent for surgical management.

### Reference Outcome Measure

The reference measure used for convergent validity was the Lower Extremity Functional Scale (LEFS) [12], which was completed prior to surgery and after surgery along with the VPM. The LEFS is a validated, patient-reported outcome measure with 20 questions, with a score ranging from 0-80, where higher numbers indicate better function. The LEFS has shown validity and reliability in patients with lower-extremity osteoarthritis and after hip and knee arthroplasty [6,7,9,10,12,13].

### Statistical Analysis

Descriptive analyses of patient characteristics were conducted. The preoperative LEFS and VPM scores were compared with postoperative scores, and the magnitude of change over time was examined using paired, 1-tailed *t* test statistics. The analysis of covariance was used to examine the role of age, sex, and severity score on postoperative VPM scores after adjusting for the preoperative level of functional difficulty. Interactions among independent variables were explored.

Longitudinal construct convergent validity was used to examine the correlation between postoperative scores and the amount of change (*postoperative score – preoperative score*) between the VPM and LEFS, using Spearman correlations. We hypothesized moderate correlations (*r*>0.30 and <0.50) [14] between the VPM total score and the LEFS score due to using different modes of measurement (self-report survey vs video-based survey).

Known-groups validity in a longitudinal context is examined by the assessment of average change in subgroups that are expected to have experienced a different amount of change in function after surgery. The receive operating characteristic curve and the area under the curve (AUC) value (area under the receiver operating characteristic curve) were used to assess the diagnostic ability of the VPM in detecting a difference in change over time between a binary predictor that indicated priority for surgery (eg, severity score ≥7 and <7). An AUC of above 0.70 is considered sufficient for diagnostic validity in clinical practice [15].

Sensitivity to change refers to the capacity of a tool to measure the magnitude of observed change statistically. We therefore measured the magnitude of change between the scores at baseline and following surgery using the standardized response mean (SRM). The SRM was calculated as *(mean change score / SD of the change score)* [14]. The Cohen criteria were used to interpret the magnitude of the SRM value [16]. An SRM of 0.80 is considered large in clinical practice.

## Results

The data of 120 patients (n=80, 67% female and n=40, 33% male; mean age 67, SD 9 years; age range 40-90 years; n=58, 48% hip osteoarthritis and n=62, 52% knee osteoarthritis) were examined. Table 1 shows the demographic characteristics of the sample included. There was a statistically significant improvement in both LEFS (*t*_119_=16.04, *P*<.001) and VPM total scores (*t*_119_=13.92, *P*<.001). Younger age (*F*_4,115_=9.93; *P*=.002) and male sex (*F*_4,115_=4.68; *P*=.03) were predictive of better VPM scores at follow-up after adjusting for the preoperative VPM score and severity of the condition.

The results of the longitudinal convergent validation showed strong correlations between the postoperative LEFS and VPM scores (*r*=0.66; *P*<.001) and moderate correlation between the change scores of these measures (*r*=0.51; *P*<.001).

**Figure 1:**
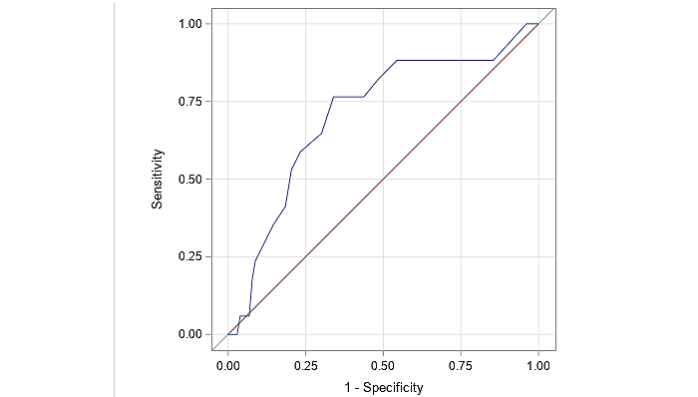
ROC analysis of the VPM change score in relation to surgical urgency. ROC: receiver operating characteristic; VPM: Virtual Performance Measure. Model (0.7079), ROC1 (0.5000).

The logistic regression that calculated the overall diagnostic value of the VPM change score for the differentiation between high priority versus moderate or low priority (based on the SSS) showed acceptable predictive validity, with an AUC of 0.71 (95% CI 0.57-0.84; Figure 1).

The SRM was 1.27 (95% CI 1.09-1.45), indicating good sensitivity to change and ability of the VPM tool to detect a true effect of surgical management.

**Table 1:** Demographics, disease characteristics, and preoperative and postoperative outcomes (N=120).

Variables		Values
Age (years), mean (SD; range)		67 (9; 40-92)
**Sex, n (%)**		
	Female	80 (67)
	Male	40 (37)
**Affected joint, n (%)**		
	Hip	58 (48)
	Knee	62 (52)
**Walking devices, n (%)**		
	Yes	35 (29)
	No	85 (71)
**Gait, n (%)**		
	Normal	10 (8)
	Abnormal	103 (86)
	Missing	7 (6)
**Severity score, mean (SD)**		
	Clinical (0-3)	2 (0.48)
	Functional (0-3)	1.90 (0.50)
	Radiological (0-2)	1.85 (0.34)
	Total score (0-8)	5.8 (10.96)
**Outcome measures, mean (SD)**		
	Preoperative LEFS^a^ score	32 (14)
	Postoperative LEFS score	59 (13)
	Preoperative VPM^b^ score	60 (18)
	Postoperative VPM score	81 (13)

^a^LEFS: Lower Extremity Functional Scale.

^b^VPM: Virtual Performance Measure.

## Discussion

### Principal Findings

This study demonstrated longitudinal convergent construct and known-groups validity as well as high sensitivity to change in patients with osteoarthritis of the hip and knee joints who had undergone joint replacement surgery. The postoperative and change scores of the VPM correlated strongly with those of the LEFS, a self-report functional tool. The VPM score was able to differentiate between patients who were considered urgent versus nonurgent for surgery (high priority vs moderate or low priority) based on the severity score. Younger patients and male patients showed a better postoperative VPM score after accounting for the preoperative VPM score and severity of the condition.

The VPM has shown cross-sectional convergent and known-groups validity in patients with knee and hip osteoarthritis [6,7]. The cross-sectional validity, however, is not sufficient for using a tool after an intervention, and longitudinal studies are required to detect change by extending beyond a single point in time and establishing the impact of surgery on improving function. To our knowledge, this is the first study that demonstrates the longitudinal validity of a digitally based outcome measure that uses a human model. In the cross-sectional study of patients with knee osteoarthritis [6] and hip joint osteoarthritis [7], the correlations between the preoperative LEFS and VPM score were 0.66 (*P*<.001) and 0.68 (*P*<.001), respectively. In this study, there was a similar correlation between the postoperative scores (*r*=0.66; *P*<.001). The correlation between the change scores of the LEFS and VPM was lower (*r*=0.51; *P*<.001). In terms of known-groups validity, AUC values of 0.90 and 0.72 were reported in relation to candidacy for total knee and hip arthroplasty [6,7]. The lower AUC value of 0.71 of this study may be related to a higher variability in the patient sample and the stricter criteria of the predictor variable (urgency vs candidacy).

The sudden rise in popularity and demand for virtual care was a consequence of the 2020 COVID-19 pandemic, which required a different approach to health care utilization and access across the world. In the postpandemic era, clinicians continue to rely on virtual modes of examination and management to maintain efficiency and access. Thus, establishing the diagnostic validity of digital tools ensures the accuracy of clinical information obtained through virtual care. Improved access to broadband internet in rural and remote areas and higher use of new technologies by older individuals will lessen the burden of commute for families who often have to drive their older family members to arthroplasty centers, which are often located in larger cities. As technology evolves, alternative approaches to virtual functional assessment that provide accurate data on range of motion and function should be explored in osteoarthritis care. A potential tool for future virtual care is the sensorless motion tracking technology that uses cameras and computer vision software to track the movements of a patient on personal devices such as a phone, tablet, or laptop. With the use of artificial intelligence, sensorless tracking systems may provide accurate and reliable results with no additional hardware; this avoids the complexity of sensor-based systems that require wearing multiple high-cost sensors and devices and have limited ability to track related body parts, thus affecting calibration and scaling.

The implication of this study in the era of fast developing new technologies is establishing confidence in the ability of a digital tool to provide valid results, predict true change, and differentiate between groups with different characteristics and patterns of recovery, while improving access. These results will inform further research in more diverse populations, shape policy, and help to find solutions to today’s health care challenges.

This study has some limitations. Patients who participated in this study had advanced osteoarthritis that required joint arthroplasty. The results may not be applicable to patients with less pathology and higher functional levels. The model used in the videos may not represent younger patients, female patients, or patients with greater levels of obesity, and these potential differences warrant investigation in future studies.

### Conclusions

The VPM demonstrated sufficient longitudinal convergent and known-group validity as well as sensitivity to change in patients with hip and knee osteoarthritis following arthroplasty. This tool has the potential to improve the delivery of care by increasing access, reducing the frequency of in-person visits, and improving the overall efficiency of the health care system following a major surgery.

## Abbreviations

AUC: area under the curve
LEFS: Lower Extremity Functional Score
SRM: standardized response mean
SSS: Severity Scoring System
VPM: Virtual Performance Measure

## Data Availability

Due to ongoing nature of the study, partial data are available from the corresponding author on reasonable request.
